# Two Instances of the Good Effects of Blisters in Incontinence of Urine

**Published:** 1786

**Authors:** Isaac Oliphant

**Affiliations:** Surgeon in London


VII. *Two Instances of the good Effects of Blisters in Incontinence of Urine. Communicated in a Letter to Dr.* Simmons *by Mr.* Isaac Oliphant, *Surgeon in London.*

HAVING experienced, in two instances of incontinence of urine, the efficacy of the mode of treatment recommended by the late Dr. Dickson in this complaint, I beg leave to communicate to you the following account of the cases, and request you to lay it, if you think proper, before the public.

The

The ſubject of the firſt caſe was Elizabeth Mitchel, a girl fourteen years of age, who had been ſubject to an involuntary diſcharge of urine, while aſleep, ever ſince ſhe could recollect any thing. This failing, as ſhe was a cleanly, induſtrious girl, gave her a great deal of uneaſineſs.

She applied to me for relief November 5th, 1783: ſhe was then hale, and of a healthy complexion, but had not yet menſtruated. A bliſter was ordered to be applied to the lower part of the ſacrum, and ten grains of olibanum, with the ſame quantity of extract of bark, to be taken twice a day.

Dreading the effect of the bliſter, ſhe at firſt only took the medicines; but receiving no benefit from it in the courſe of eight or ten days, I prevailed on her to apply the bliſter, and to keep it on till full effect was procured.

From nearly the firſt operation of the bliſter the *acceleratores* were inflamed, and ſhe obſerved a little capacity in their retentive power during ſleep, as ſhe awoke with a quantity of urine in her bladder for a voluntary expulſion, as in the day time, or when awake. This effect gradually improved, from day to day, till ſhe was perfectly well, on December the 2d

 followfollowing, and has continued ever ſince free from the diſeaſe. She did not menſtruate during this period, neither were there any ſigns of the approach of the menſes till twelve months after.

Sarah Clarke, of the pariſh of St. James's, aged nine years, laboured under a ſimilar complaint for ten months. She was treated as above, and with the ſame happy conſequences. She was well in about a fortnight.

Theſe caſes appeared to me to ariſe from a defect of nervous influence, or a want of balance between the expulſive and retentive powers of the bladder, as it never had taken place in the one caſe without the aid of the will, and in the other it was equally ſuſpected to be deficient from morbid cauſes. The reſult ſeems to corroborate this idea.

*Broad Street, Soho*,

Nov. 8, 1785.

